# Innovative method for rapid detection of falsified COVID-19 vaccines through unopened vials using handheld Spatially Offset Raman Spectroscopy (SORS)

**DOI:** 10.1016/j.vaccine.2023.10.012

**Published:** 2023-10-20

**Authors:** Sara Mosca, Qianqi Lin, Robert Stokes, Tehmina Bharucha, Bevin Gangadharan, Rebecca Clarke, Laura Gomez Fernandez, Michael Deats, John Walsby-Tickle, Benediktus Yohan Arman, Shrikrishna R. Chunekar, Kundan D. Patil, Sunil Gairola, Kerlijn Van Assche, Susanna Dunachie, Hamid A. Merchant, Rutendo Kuwana, Alexandrine Maes, James McCullagh, Céline Caillet, Nicole Zitzmann, Paul N. Newton, Pavel Matousek

**Affiliations:** a Central Laser Facility, Research Complex at Harwell, STFC Rutherford Appleton Laboratory, UKRI, Harwell Campus, OX11 0QX, UK; b Agilent Technologies LDA UK, Becquerel Avenue, Didcot OX11 0RA, UK; c Department of Biochemistry, University of Oxford, Oxford OX1 3QU, UK; d Kavli Institute for Nanoscience Discovery, University of Oxford, Oxford OX1 3QU, UK; e Department of Chemistry, University of Oxford, Oxford OX1 3TA, UK; f Medicine Quality Research Group, NDM Centre for Global Health Research, Nuffield Department of Medicine, University of Oxford, Oxford OX3 7LG, UK; g Mahidol-Oxford Tropical Medicine Research Unit, Faculty of Tropical Medicine, Mahidol University, Bangkok 10400, Thailand; h Infectious Diseases Data Observatory, Centre of Tropical Medicine & Global Health, Nuffield Department of Medicine, University of Oxford, Oxford OX3 7LG, UK; i Serum Institute of India Pvt. Ltd., 212/2, Hadapsar, Pune 411028, India; j Department of Microbiology and Infectious Diseases, Oxford University Hospitals NHS Foundation Trust, Oxford OX3 9DU, UK; k NIHR Oxford Biomedical Research Centre, Oxford University Hospitals NHS Foundation Trust, Oxford OX3 9DU, UK; l Department of Pharmacy, School of Applied Sciences, University of Huddersfield, Queensgate, Huddersfield HD1 3DH, UK; m Regulation and Safety Unit, Regulation and Prequalification Department, Access to Medicines and Health Products Division, World Health Organization (WHO), Geneva, Switzerland

**Keywords:** Spatially Offset Raman Spectroscopy, Handheld, COVID-19 vaccines, Non-invasive, Falsified, Counterfeit, Supply chain

## Abstract

Preventing, detecting, and responding to substandard and falsified vaccines is of critical importance for ensuring the safety, efficacy, and public trust in vaccines. This is of heightened importance in context of public health crisis, such as the COVID-19 pandemic, in which extreme world-wide shortages of vaccines provided a fertile ground for exploitation by falsifiers. Here, a proof-of-concept study explored the feasibility of using a handheld Spatially Offset Raman Spectroscopy (SORS) device to authenticate COVID-19 vaccines through rapid analysis of unopened vaccine vials. The results show that SORS can verify the chemical identity of dominant excipients non-invasively through vaccine vial walls. The ability of SORS to identify potentially falsified COVID-19 vaccines was demonstrated by measurement of surrogates for falsified vaccines contained in vaccine vials. In all cases studied, the SORS technique was able to differentiate between surrogate samples from the genuine COVISHIELD™ vaccine. The genuine vaccines tested included samples from six batches across two manufacturing sites to account for any potential variations between batches or manufacturing sites. Batch and manufacturing site variations were insignificant. In conjunction with existing security features, for example on labels and packaging, SORS provided an intrinsic molecular fingerprint of the dominant excipients of the vaccines. The technique could be extended to other COVID-19 and non-COVID-19 vaccines, as well as other liquid medicines. As handheld and portable SORS devices are commercially available and widely used for other purposes, such as airport security, they are rapidly deployable non-invasive screening tools for vaccine authentication.

## 1 Introduction

Vaccines are vital and cost-effective public health interventions that prevent community transmission and reduce the risks of severe infectious diseases caused by multiple pathogens. Their critical importance has been highlighted most recently by the COVID-19 pandemic. The health and economies of the world’s population and recovery from COVID-19 depend on effective, safe, accessible, and affordable vaccines. Before the COVID-19 pandemic there were numerous reports of falsified vaccines used for other infectious diseases [1,2]. Such occurrences undermine the reputation of vaccines, and by consequence the effective implementation of immunization programs. Public trust and vaccine uptake are threatened with associated increased morbidity, mortality, and economic burden. COVID-19 disrupted markets and overwhelmed the capacity of regulatory authorities. Coupled with unprecedented global demand, time pressure and inequitable distribution, this created vulnerabilities and opportunities for falsifiers to infiltrate vaccine supply and distribution chains leading to an increased risk of falsified products reaching patients. Falsifiers may obtain or copy genuine vaccine vials and fill them with non-vaccine liquids (e.g., saline or antibiotics), produced in non-sterile conditions risking public harm. With the unprecedented volume of COVID-19 vaccines produced and distributed worldwide, vaccine falsification and subsequent risks to public safety are a global challenge. During the COVID-19 pandemic a large number of falsified COVID-19 vaccines were reported [3], unfortunately these were almost always identified after they had been already used by human subjects. This evidences the problem and the societal impact of our inability to rapidly detect falsified products in the supply chain. Some falsified products that were intercepted were reported to only contain saline solution (China [4] and India - Mumbai [5]), in other cases amikacin (India - Kolkata) [6], glucose (Philippines) [7] and hyaluronic acid (Poland) [8] were found.

Without effective safety measures and analytical tools that can be easily deployed in the field, falsified medicines and vaccines may not be detected promptly before reaching patients. To address this global health issue, WHO Member States adopted a PREVENT, DETECT, and RESPOND strategy [9]. However, the detection of falsified vaccines using reference assays in the field is complicated as these tests can only be performed in a few specialized laboratories [10]. Barcode tracking and authentication systems (aka serialisation) are being deployed but rapid, global rollout is an enormous task, particularly in low-resource settings. There are no accessible systems with rapid turnaround for vaccine identification, and minimal published research on accessible, ideally portable, and non-destructive devices to screen vaccines within supply chains to find suspected stocks to be sent for confirmatory tests in reference laboratories. To date, research of effective screening devices has focused mainly on those aimed at testing tablets/capsules [11,12], with limited attention to vaccines. Recently, NIR (Near-infrared) absorption spectroscopy was demonstrated in a proof-of-concept study that it is a viable tool for screening vaccine solutions through unopened vials by monitoring high overtones and combination vibrational modes of the solution [13]. In this study, the capability of the NIR to discriminate genuine products from surrogates for falsified products was not explicitly investigated. The NIR instrument used was a benchtop rather than a handheld device making its field deployment more challenging. An innovative approach, therefore, is urgently needed to find methods for detecting falsified vaccines directly within supply chains to protect the global COVID-19 vaccine supply, as well as for other vaccines and essential liquid medicines that are at an elevated risk of falsification.

Here we investigate the potential of the recently developed Spatially Offset Raman Spectroscopy (SORS) [14] technology. Raman spectroscopy is a vibrational spectroscopy conveying rich chemical information on molecules present in samples through detected inelastically scattered light. SORS enables analysis through containers, both clear (aka transparent) and diffusely scattering (aka opaque), with enhanced sensitivity compared to conventional Raman spectroscopy by supressing interfering signal emanating from vial wall – in line with an earlier study [15]. It uses a physical separation (spatial offset) between collection and illumination zones on the sample surface to suppress interfering signals (both Raman and fluorescence) from the surface layer (e.g., container wall) in order to more clearly reveal the sub-surface Raman signature (e. g., container contents). It is widely used, for example in pharmaceutical manufacture, in raw material identification through packaging and containers, in airport security to screen liquids taken on board in planes, and in the detection of explosives, narcotics and hazardous materials in the field using handheld devices [16]. SORS has also been demonstrated as a potential tool to intercept falsified solid oral dosage forms of pharmaceuticals [17,18]. To the best of our knowledge, SORS has not been investigated yet for the analysis of vaccines. Vaccines in particular, represent a non-trivial analytical challenge, due to a very low concentration of vaccine ingredients in a backdrop of intense fluorescence signals from containers, such as glass vials that are commonly used in vaccines and other injectables. A previously reported investigation of formulated vaccines using Raman spectroscopy involved invasive analysis only [19]. Here, we focus specifically on non-invasive analysis performed through manufacturer-sealed containers to simulate anticipated usage in-field by operators without needing specialist analytical laboratory skills. SORS analysis does not require opening of vials or syringes and allows further use of the probed samples, preventing wastage. This enables a larger fraction of vaccines to be analysed in a rapid risk-based post-market surveillance, without the loss of precious vaccines.

## 2 Experimental

### 2.1 SORS handheld device Resolve

**Fig. 1:**
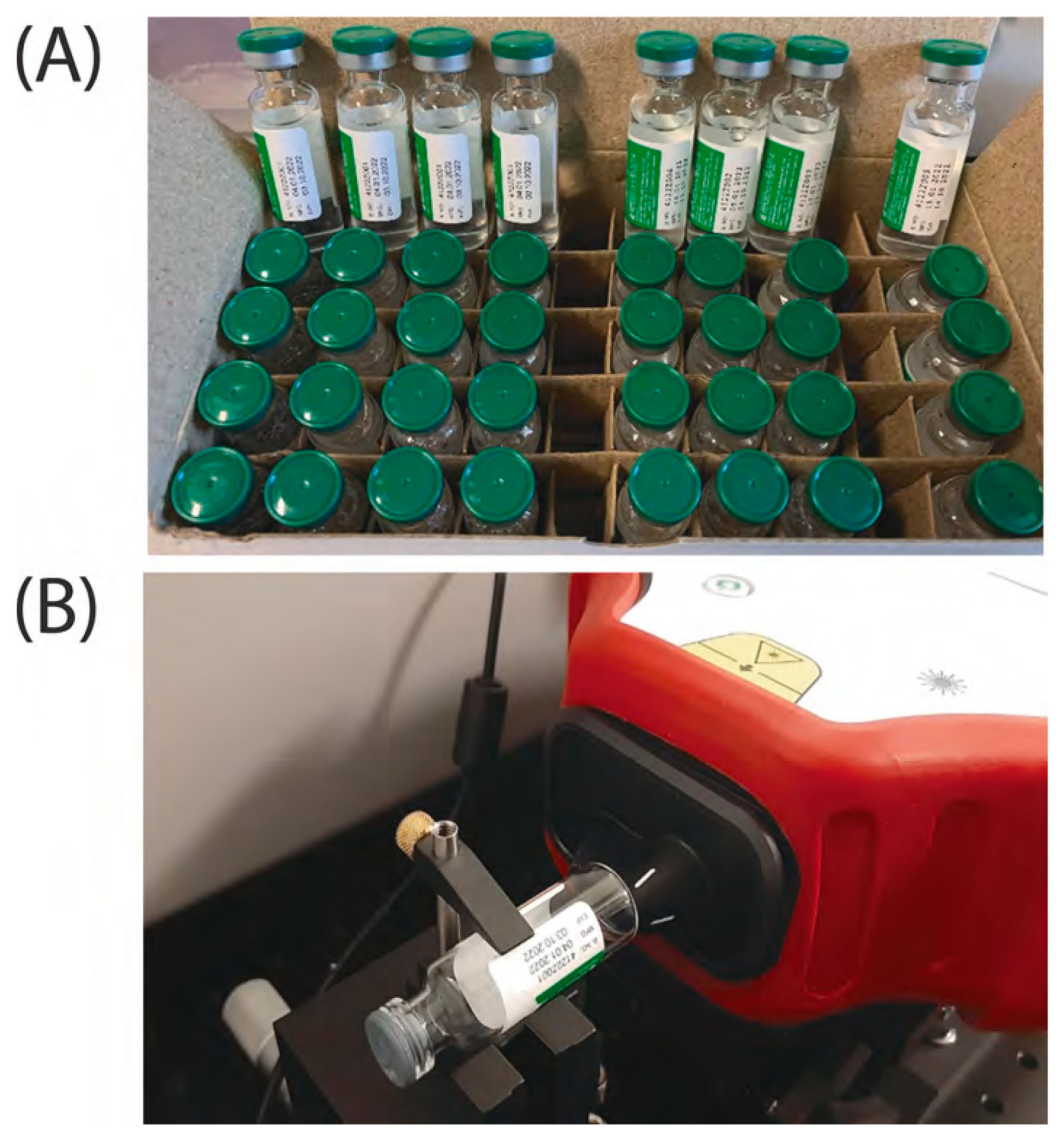
(A) SII COVID-19 vaccine samples in distribution (glass) vials and (B) the horizontal positioning of the vials in front of the SORS handheld instrument *Resolve*.

The measurements were performed using a handheld commercial SORS instrument (*Resolve*, Agilent Technologies, Oxfordshire, UK). The spectra were collected with 25 s (zero spatial offset = 1 s × 5 a (acquisitions); spatial offset = 2 s × 10 a) overall acquisition time using a laser with the 830 nm excitation wavelength at the power of 475 mW. SORS spatial offset was set to 5.5 mm (the overall measurement time accounting for automated instrumental calibration and background measurements was approximately 1.5 min). The measurements were performed in a dark laboratory. For future field deployment, a dedicated light proof compartment tailored to vaccine vials can be readily constructed by the manufacturer. A similar compartment already exists as an instrumental accessory for “*Resolve*” for processing other types of sample containers. The vials were measured lying on their side, scanned through their bottom (see Fig. 1). This orientation was adopted for consistency with other potential vaccine formulations comprising occasionally also partially filled vials and to avoid labels which can stretch across the entire side wall of vials in some commercially available vaccine products. Six measurements at different vial positions were acquired from each vial.

### 2.2 Samples

The COVID-19 vaccine samples (45 vials in total) were kindly provided by the Serum Institute of India (SII, COVISHIELD™), Pune, India (see Fig. 1) formulated as the final product. No additional quality control was conducted by us. These were vaccine formulations packaged in their distribution vials (10 dose vials) as per Table 1. The glass vials were of an external glass diameter of 15 mm and their height was 52 mm without the stopper (54 mm with the stopper). Five batches were produced at the Hadapsar factory and one batch at the Manjari factory, both located in Pune, Maharashtra, India. All samples were initially measured well-within their date of expiry. Subsequent control measurements of surrogates of falsified vaccines were performed in conjunction with a subset of the genuine products (described in Table 1) to assure correct instrumental performance. At this point, these control genuine samples were out of date but still within 1-month post-expiry and were kept as per prescribed cold storage with no evidence of adverse impact on the analytical results presented in this study.

The precise concentrations of vaccine excipients in formulations have been excluded from this manuscript on global public health security grounds.

#### 2.2.1 Genuine vaccines and single solutions of dominant excipients

For the comparative vaccine excipient measurements, the following solutions were used: Water for injection (Ultrapure water (UPW)), ethanol in water (<1 % v/v; Fisher Scientific), sucrose (<10 % in distilled water), L-histidine in water (<10 mM; Sigma-Aldrich). Although not necessarily identical these were similar to those used in the authentic vaccines.

#### 2.2.2 Surrogate falsified vaccines

Surrogates for potential and intercepted falsified vaccines (identified during the COVID-19 pandemic and before) were prepared and placed into empty SII distribution vials (secured by emptying SII COVID-19 vaccine vials) apart from hyaluronic acid, amikacin and gentamicin which were received in glass sealed vials and as such measured through their native glass vials. This decision was supported by the fact that both hyaluronic acid and amikacin were reported to be intercepted in their native vials which were different from those of genuine vaccines [20,21]. Up to six SORS spectra from each surrogate sample were obtained and processed identically to the genuine vaccine samples. The formulations for these are shown in Table 2.

#### 2.2.3 Degraded samples

Three heat-degraded samples were also prepared for assessing the potential impact of temperature excursions in supply chains on SORS spectra. The first sample was prepared by taking a genuine vaccine sample and subjecting it to an elevated temperature of 45 °C for exactly 1 week. The second sample was prepared by subjecting a vaccine vial to three freeze/thaw cycles. In each cycle, it was frozen at −80 °C for 24 h, and thawed at room temperature (19 °C) for 1 h. The third sample was prepared by submerging a vaccine vial in boiling water for 10 min to induce extensive thermal degradation.

### 2.3 Data processing

The Raman spectra were processed manually by exporting them from the device to a computer. They were analysed using a semi-automated SORS container/content signal separation routine involving basic scaled subtraction of the zero spatial offset spectrum from the spatially offset spectrum. The scaling factor was selected to bring the glass fluorescence around its maximum (at ~675 cm^−1^) approximately to zero in the subtracted spectrum [15]. This revealed a pure Raman spectrum of the vial content. The code was written in Python and also utilised sub-sequent removal of residual background profile using 5th order polynomial subtraction.

The Raman spectra of vaccines and surrogates for falsified vaccines were first truncated below 760 cm^−1^ and above 1720 cm^−1^. Then the residual background spectral profile was removed again (5th order polynomial) and the spectra were normalised (standard normal variate, SNV) and mean-centred apart from the degradation sample set where the same pre-processing was used but without the mean centering. Principal Component Analysis (PCA) was then performed on the resulting spectral data (Solo, Eigenvector Research Inc.). One outlier (of unknown origin) was removed from the genuine data set of 50 spectra (each vial was measured once, then 5 additional repeat measurements were performed on another day along with measuring vaccine surrogates for falsified products).

## 3 Results and discussion

### 3.1 Vaccine constituents identification by SORS

**Fig. 2:**
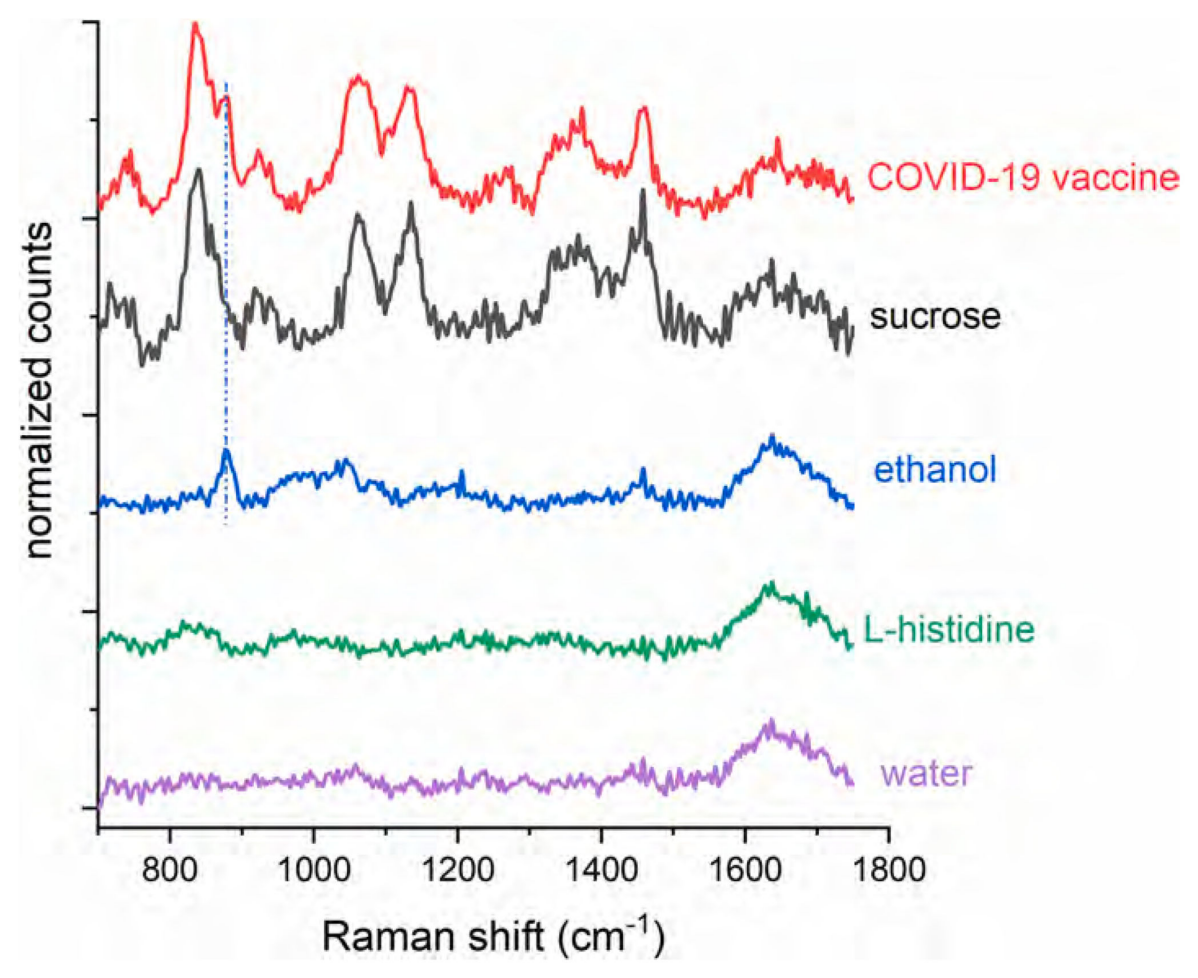
Representative Raman spectra of the SII COVID-19 vaccine solution along with solutions of individual major excipients of this vaccine formulation measured using the handheld *Resolve*.

In the first set of experiments we set to understand which chemical constituents contribute to the observed SORS vaccine spectra. Fig. 2 compares the SORS spectra for the SII COVID-19 vaccine and separate individual excipients when measured through the genuine SII supplied glass vials. Both sucrose and ethanol (~880 cm^−1^) constitute the main part of the observed vaccine Raman spectrum. This is expected as these were the two dominant excipients. Another clearly visible component is the Raman band of water at ~1640 cm^−1^, which correlates well with the pure spectrum of water. This is a very important feature of the detected spectrum as it enables the multivariate analysis to also capture the relative concentration of the observable excipients in the vaccine spectrum relative to the dominant matrix, water. It should be noted that another major component of the vaccine samples, and generally also of other non-COVID-19 vaccine solutions, is sodium chloride (the solute in the saline solution) which is present in aqueous environments as individual ions (Na^+^ and Cl^-^). Due to its dissociation by solvation into single atomic entities, it does not exhibit any ‘intramolecular’ vibrational motions and therefore does not give rise to any Raman features in the spectrum.

The component with the next highest concentration in the SII COVID-19 vaccine solution is L-histidine. No significant SORS features were detected through vials for this excipient, neither for the vaccine nor the pure L-histidine solution alone (residual peaks that are present aside from the 1640 cm^−1^ water band are thought to originate from imperfect subtraction of the large fluorescence background from the glass vial; most were also noted in the positive control water Raman spectrum).

### 3.2 Differentiation of genuine vaccines from surrogates for falsified solutions

**Fig. 3:**
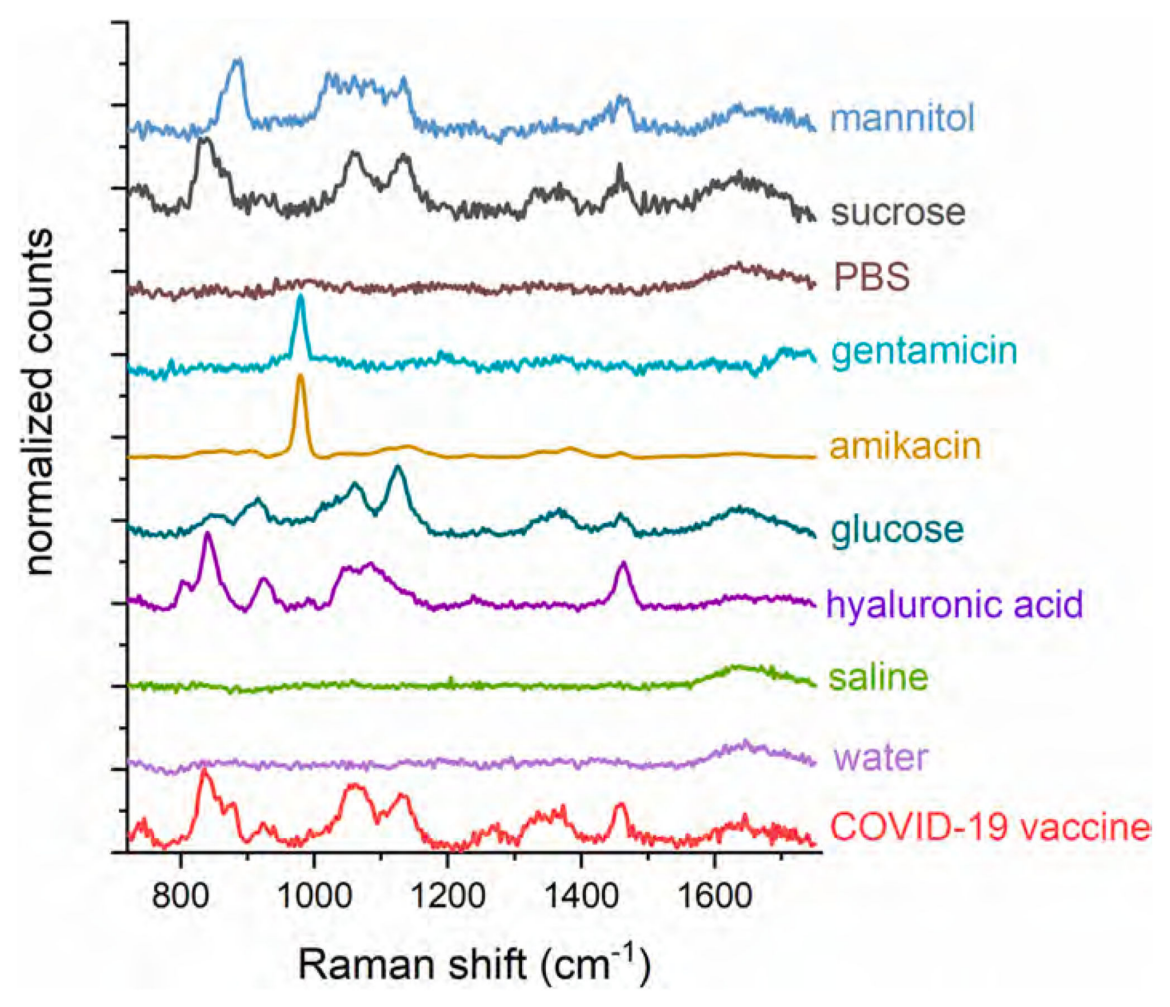
Representative Raman spectra obtained on the handheld *Resolve* instruments of a genuine COVID-19 vaccine solution (the bottom spectrum) along with surrogates for falsified COVID-19 products and excipients commonly used in other vaccines. All the spectra were obtained by measurements through original SII glass vials using SORS.

**Fig. 4:**
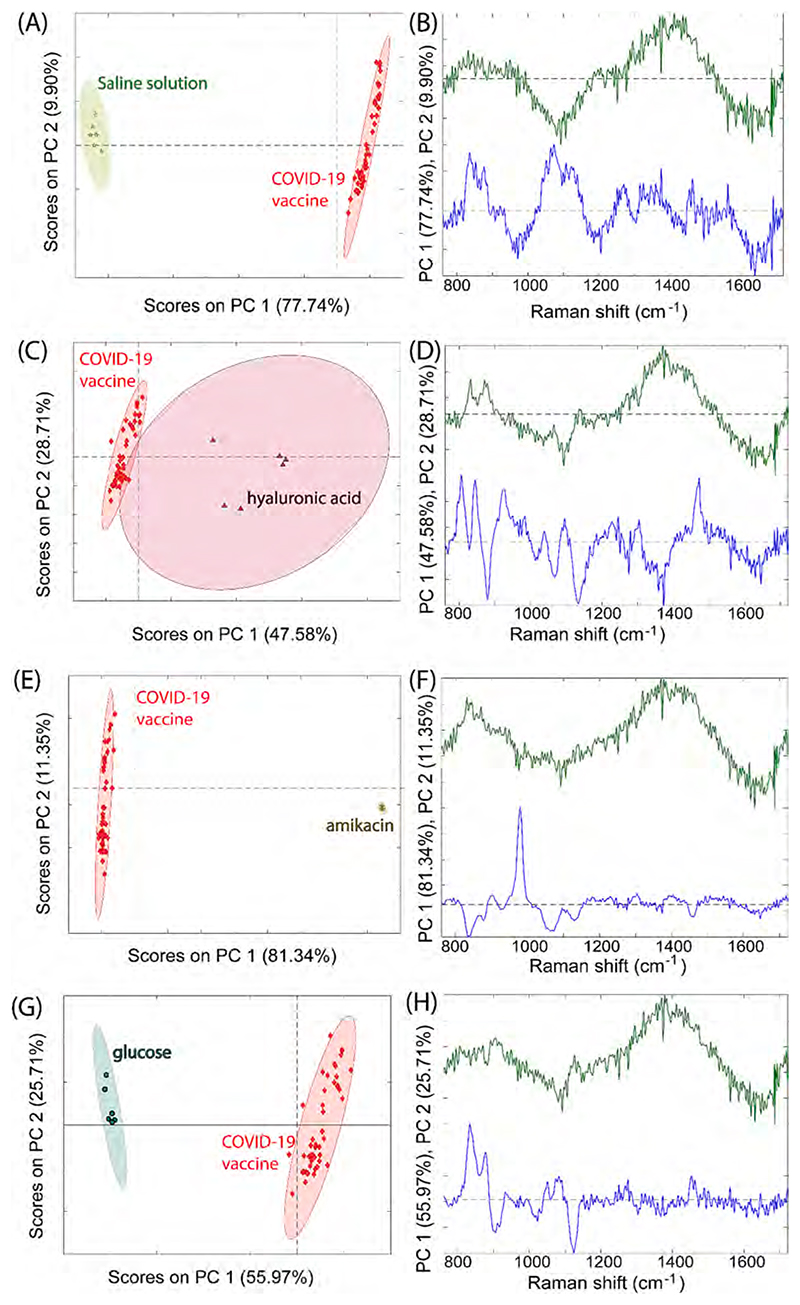
(A, C, E, G) PCA score plots of the two most significant principal components showing the ability to discriminate the genuine COVID-19 vaccines from the surrogate falsified for intercepted falsified COVID-19 vaccines held in a genuine vaccine vial and measured through vials by SORS. The ellipsoids plotted in the PCA score plots represents 95% confidence intervals for each class. (B, D, F, H) The relevant eigenvectors are also shown evidencing that the discrimination between samples is based on chemical information contained in spectra rather than interfering fluorescence or other artefacts of measurement.

The Raman spectra shown in Fig. 3 exhibited distinctly different spectral features, suggesting the possibility of differentiating them by automated means, thus with easy readability for the end-user with no or limited chemometrics knowledge. This differentiation, performed by PCA, is illustrated in Fig. 4 where the PCA score plots of the first two most significant principal components are shown along with the corresponding eigenvectors for surrogates of actually intercepted falsified products in COVID-19 supply chains. These results showed a good degree of separation of surrogates for falsified products from genuine COVID-19 vaccines. In Fig. S1 we further supplied the discrimination attained using different surrogates for other potentially falsified products of COVID-19 vaccines that have not yet been observed in COVID-19 supply chains. Again, good separation was achieved for all the potential constituents of falsified products investigated in this study. A good degree of discrimination was achieved even for sucrose solution, which is a major excipient in the original vaccines itself, making the differentiation more challenging. This was achieved, to a great degree, by the presence of a characteristic Raman feature at ~880 cm^−1^ in the genuine vaccine solutions which was absent in the surrogate as seen in the eigenvector plots. To test the differentiation capability we performed PLS-DA calculations (Partial Least Squares - Discriminant Analysis) on genuine versus individual falsified formulations. The results are summarised in Supplementary Information section (SI) in Table S1. Notably, sensitivity and specificity of cross validation for detecting falsified vaccines was 100 % for all the surrogates for falsified formulations apart from sucrose, in line with their distinctly different chemical composition detected by SORS. The sensitivity of the differentiation of sucrose from the genuine product dropped to 83 % reflecting the similarity in the formulations (the specificity remained 100 %).

A further study was carried out on thermal degradation yielding no statistically significant features attributable to appearance or disappearance of chemical (vibrational) species in vaccine formulations. This is summarised in Supplementary Information (SI) (Fig. S2).

Conventional (non-SORS based) Raman instruments, such as the *TruScan* (Thermo Fisher) are also used in the detection of substandard and falsified solid dosage formulation medicines [11,12]. We therefore also investigated the use of the *TruScan*. The details are given in the SI. In summary, the tests showed that conventional Raman spectroscopy is also capable of yielding well defined, low photon shot noise vaccine Raman signatures noninvasively and as such it also constitutes a potentially viable tool for differentiating falsified vaccines from genuine products in the field. Although this is with retaining a significant interfering fluorescence background originating from vaccine glass vial [23]. These are believed to originate from glass impurities and could pose a challenge when applying to vials made of different types of glass or of different manufacturing batches of glass.

These observations confirm the viability of using the intrinsic molecular signature of several excipients within vaccines for the authentication of vaccine products using Raman spectroscopy through unopened vials. Both the chemical identity as well as their concentration, which are typically highly controlled in the manufacturing process of original vaccines, provides controlled parameters that could be tightly monitored. In conjunction with existing security features, such as those on vaccine labels and packaging, SORS could be used to identify falsified products in vaccine distribution chains. Any suspected stock found through initial SORS screening could then be passed on to a reference laboratory for confirmatory testing using established, highly sensitive but invasive, chromatographic, and mass spectrometric techniques specially optimised for the purpose.

Manual data processing was employed for this proof-of-concept study. However, spectra of similar high quality in a fully automated process in the field is also possible following improvements in the handheld device, for instance, sample presentation (by providing a dedicated light-tight sample holder for small vials) and enhanced data processing using the existing in-use automated software (e.g. code optimised for the low concentration measurements).

The non-invasive nature of the analysis enables scanning of large quantities of vaccine samples at various points of supply chains (e.g., major countries import hubs) while retaining samples for example for patients or for further analysis when needed. The technique, therefore, can be a preventive scanning or scanning for already suspected shipments based on intelligence or other notable observations or suspicious defects in vaccine labels and packagings.

### 3.3 Batch dependence

**Fig. 5:**
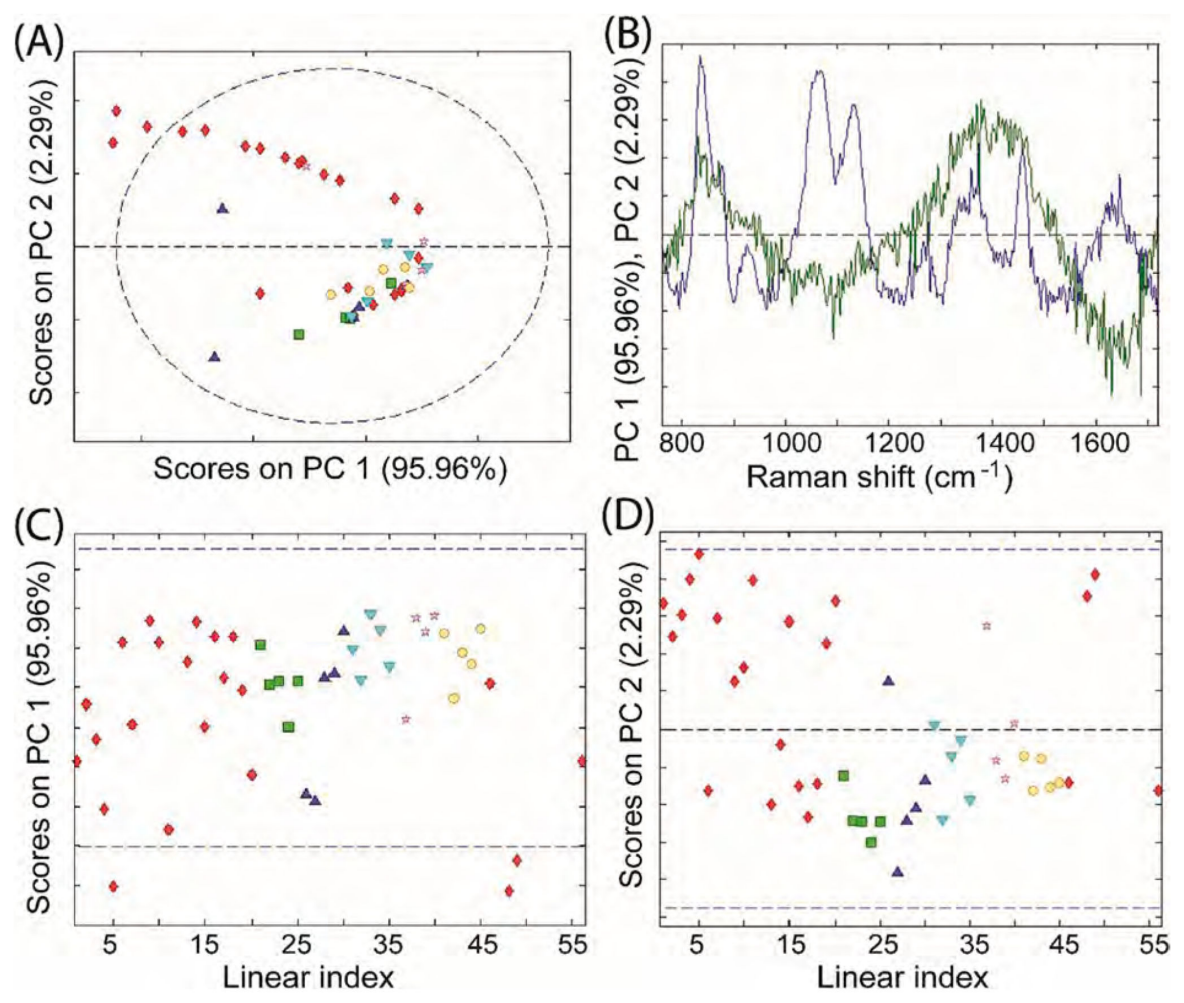
(A) PCA score plot for genuine SII COVID-19 vaccine SORS spectra measured non-invasively through originally supplied vaccine glass vials. Different symbols indicate different batches with the red diamond designating the largest batch (Batch 1). The other data points (green squares, light blue upside-down triangles, dark blue triangles, pink stars) are of batches from the same manufacturing site except yellow circles (Batch 6) which represent a batch of five vials from a different manufacturing site. (B) The relevant eigenvectors are also shown along (C, D) with their scores. (For interpretation of the references to colour in this figure legend, the reader is referred to the web version of this article.)

Batch and manufacturing site variations were also investigated. No significant batch or site variations were observed in the collected SORS data (Fig. 5). Although some variations were present, these appear random in nature and did not carry any significant molecular (vibrational) signatures. It is suspected that the observed variance is possibly caused by sample repositioning and instrumental influences as evident from PCA components and the measurement time sequence. This batch insensitivity makes the deployment of the technique in the field for the interception of falsified vaccine products easier, as reference spectra from one batch can be used with products of other batches and manufacturing sites without the loss of differentiation capability of falsified products.

## 4 Conclusions

Using the Serum Institute of India’s COVISHIELD™ COVID-19 vaccine, we have demonstrated the potential of portable SORS to qualitatively analyse vaccine solutions non-invasively through commercially distributed sealed glass vials. The results indicate that SORS can detect the chemical identity of dominant vaccine ingredients. Together, SORS provides an intrinsic molecular fingerprint signature of the internal components of the vaccine and therefore can be an invaluable tool to discriminate original vaccine products from those falsified.

Moreover, no statistically significant batch or manufacturing site variability was identified which will make the deployment of the technology more straightforward. The thermal degradation study also did not yield any significant chemical alterations within the formulation that could interfere with the identification of genuine from falsified constituents. However, it is worth noting that the technique is not suitable for detection and monitoring of degradation related to this type of vaccine (for instance detecting degradation due to temperature excursions in supply chain)) similar to those studied here. Also the technique cannot identify the presence of vaccine active ingredients. Further work is needed to evaluate SORS with a wider diversity of vaccines and to further compare with conventional Raman spectroscopy. We would also like to note that we would be happy to discuss practicalities and specifics of implementation of this technology in the field, including technique’s limitations and strengths, with potentially interested organisations.

This proof-of-concept study on a rapid and non-invasive SORS technique for vaccine analysis opens the door to screening other COVID-19 and non-COVID-19 vaccines, as they often contain a diverse range of excipients at characteristic concentration levels. Portable, reliable, and robust SORS devices that can interrogate the components of vaccine excipients within originally supplied vaccine vials, with minimal operator training, are already available for other purposes. Therefore, the technique can be employed relatively easily within the distribution chains of vaccines globally. The novel technique employing SORS reported here could reduce the sizeable risk of infiltration of vaccine supply chains by falsified products, and help address this significant, yet currently largely neglected, public health concern.

## Supplementary Material

Supplementary data to this article can be found online at https://doi.org/10.1016/j.vaccine.2023.10.012.

Supplementary File

## Figures and Tables

**Table 1:** Serum Institute of India (SII) COVID-19 vaccine (COVISHIELD™) samples. Batch 1–5 from SII Hadapsar manufacturing site. Batch 6 from SII Manjari manufacturing site.

Batch numbers	Number of vials
Batch 1 *(Batch No. 4122Z001)*	20
Batch 2 *(Batch No. 4122Z002)*	5
Batch 3 *(Batch No. 4122Z003)*	5
Batch 4 *(Batch No. 4122Z004)*	5
Batch 5 *(Batch No. 4122Z005)*	5
Batch 6 *(Batch No. 4121MC180)*	5

**Table 2:** List of falsified surrogate vaccines, dominant excipients in Covishield (sucrose, ethanol, L-histidine) and other excipients in other vaccines (mannitol and PBS) used for SORS experiments.

Surrogates and excipients used in Covishield/other vaccines	Composition	Comment
Water	Water for injection alone (Demo S.A. Pharmaceutical Industry)	In-house surrogate. Taken from water for injection ampoule for parenteral human use
Saline solution	0.9 % w/v NaCl in water for injection (Demo S.A. Pharmaceutical Industry)	In-house surrogate for falsified Covid-19 vaccines intercepted in China and India (Mumbai) [3]
Hyaluronic Acid	Anti-wrinkle serum. Purchased on Amazon - Guangzhou Ailian Cosmetic Co Ltd. P/N QB/T 2660Contains: water, glycerine, propylene glycol, methylisothiazolinone, bis(hydroxmethyl) imidazolidinyl urea, iodopropynyl butylcarbamate, disodium EDTA, xanthan gum, sodium hyaluronate	Surrogate for falsified Covid-19 vaccines intercepted in Poland [3] (the precise formulation and form of intercepted hyaluronic product unknown apart from it being reported containing an anti-wrinkle formulation [21])
Amikacin	250 mg/ml amikacin sulphate, sodium citrate, sodium metabisulphite and water for injection.	Surrogate for falsified Covid-19 vaccines intercepted in India (Kolkata) [3]
	250 mg/ml Hospira P/N 05015997122159	
Gentamicin	40 mg/ml gentamicin sulphate, 1.60 mg/ml sodium metabisulfite. Demo S.A. GTIN 05208063001339	Surrogate for falsified non-COVID vaccines intercepted in Indonesia [22]
Glucose	D-glucose 5.0 % w/v. Glucose (SERVA) solution prepared in distilled water	Surrogate for falsified Covid-19 vaccines intercepted in the Philippines [3]
Sucrose	<10 % w/v sucrose in water. Sucrose (Fisher Scientific) solution prepared in distilled water	An excipient in genuine Covishield vaccine
Ethanol	<1 % v/v ethanol in water	An excipient in genuine Covishield vaccine
L-histidine	<10 mM L-histidine in water	An excipient in genuine Covishield vaccine
Mannitol	5 % w/v mannitol in water. Mannitol (Sigma-Aldrich) solution prepared in distilled water	Common stabiliser in vaccines and therefore a potential falsified vaccine surrogate
Phosphate Buffered Saline (PBS)	Phosphate Buffered Saline 1X Solution (NaCl: 137 mM, KCl: 2.7 mM, Na_2_HPO_4_: 10 mM, KH_2_PO_4_: 1.8 mM) Purchased from Sigma-Aldrich	A common buffering system (excipient used in many vaccines)

## Data Availability

The dataset supporting this paper is openly available both from Mendeley Data and eData at the STFC Research Data repository [24,25].
