# Supplementary material for: Innovative method for rapid detection of falsified COVID-19 vaccines through unopened vials using handheld Spatially Offset Raman Spectroscopy (SORS)
Source: Vaccine. Author manuscript; Available in PMC 2025 Dec 24. (PMC7618537; doi:10.1016/j.vaccine.2023.10.012)
Supplement: Supplementary File [file EMS211590-supplement-Supplementary_File.docx]

**Supplementary Material**

**Innovative Method for Rapid Detection of Falsified COVID-19 Vaccines through Unopened Vials using Handheld Spatially Offset Raman Spectroscopy (SORS)**

Sara Mosca^1^, Qianqi Lin^1^^[[1]](#footnote-1)^†, Robert Stokes^2^, Tehmina Bharucha^3,4^, Bevin Gangadharan^3,4^, Rebecca Clarke^5^, Laura Gomez Fernandez^3,4^, Michael Deats^6-8^, John Walsby-Tickle^5^, Benediktus Yohan Arman^3,4^, Shrikrishna R. Chunekar^9^, Kundan D. Patil^9^, Sunil Gairola^9^, Kerlijn Van Assche^6-8^, Susanna Dunachie^7,10,11^, Hamid A. Merchant^12^, Rutendo Kuwana^13^, Alexandrine Maes^13^, James McCullagh^5^, Céline Caillet^6-8^, Nicole Zitzmann^3,4^, Paul N. Newton^6-8 *^, Pavel Matousek^1,6-8 *^

^1^Central Laser Facility, Research Complex at Harwell, STFC Rutherford-Appleton Laboratory, UKRI, Harwell Campus, OX11 0QX, UK

^2^Agilent Technologies LDA UK, Becquerel Avenue, Didcot OX11 0RA, UK

^3^Department of Biochemistry, University of Oxford, OX1 3QU, Oxford, UK

^4^Kavli Institute for Nanoscience Discovery, University of Oxford, OX1 3QU, Oxford, UK

^5^Department of Chemistry, University of Oxford, OX1 3TA, Oxford, UK

^6^Medicine Quality Research Group, NDM Centre for Global Health Research, Nuffield Department of Medicine, University of Oxford, Oxford, OX3 7LG, UK

^7^Mahidol-Oxford Tropical Medicine Research Unit, Faculty of Tropical Medicine, Mahidol University, Bangkok, 10400, Thailand

^8^Infectious Diseases Data Observatory, Centre of Tropical Medicine & Global Health, Nuffield Department of Medicine, University of Oxford, Oxford, OX3 7LG, UK

^9^Serum Institute of India Pvt. Ltd., 212/2, Hadapsar, Pune – 411028, India

^10^Department of Microbiology and Infectious Diseases, Oxford University Hospitals NHS Foundation Trust, Oxford OX3 9DU, UK

^11^NIHR Oxford Biomedical Research Centre, Oxford University Hospitals NHS Foundation Trust, Oxford OX3 9DU, UK

^12^Department of Pharmacy, School of Applied Sciences, University of Huddersfield, Queensgate, Huddersfield, HD1 3DH, UK

^13^Regulation and Safety Unit, Regulation and Prequalification Department, Access to Medicines and Health Products Division, World Health Organization (WHO), Geneva, Switzerland

*Corresponding Authors: Pavel Matousek: [pavel.matousek@stfc.ac.uk](mailto:pavel.matousek@stfc.ac.uk) and Paul N. Newton: [Paul.Newton@tropmedres.ac](mailto:Paul.Newton@tropmedres.ac)

**-------------------------------------------------------------------------------------------------------------------------------**

**Table of content:**

S1: PCA of genuine vs. surrogates for potentially falsified products p.3

S2: Thermal Degradation p.5

S3: Comparison with Conventional Raman Spectroscopy p.7

**S1: PCA of genuine vs. surrogates for potentially falsified products**

Figure S1: (a,c,e,g,i) PCA score plots of the two most significant principal components showing discrimination of genuine COVID-19 vaccines from surrogates for potential falsified products of COVID-19 vaccines held in an identical vial and measured through vials by SORS. The ellipsoids plotted in PCA score plots represent 95% confidence intervals for each class. (b,d,f,h,j) The relevant eigenvectors are also shown versus Raman wavenumbers showing molecular discrimination.

**Table S1**: Differentiation of genuine products from surrogates for falsified products using PLS-DA analysis. Both the cross-validation (CV) sensitivity and cross validation specificity for individual surrogates are reported (Venetian blinds , w/1- split and blind thickness = 1), latent variables = 2). The analysis was performed in pairs, geniuine versus falsified set of SORS spectra measured through unopened vials (as with PCA).

| Sample | Sensitivity CV | Specificity CV |
| --- | --- | --- |
| *Water* | 1 | 1 |
| *Saline solution* | 1 | 1 |
| *Hyaluronic Acid* | 1 | 1 |
| *Amikacin* | 1 | 1 |
| *Gentamicin* | 1 | 1 |
| *Glucose* | 1 | 1 |
| *Sucrose* | 0.833 | 1 |
| *Mannitol* | 1 | 1 |
| *Phosphate Buffered Saline (PBS)* | 1 | 1 |

**S2: Thermal Degradation**

Thermally degraded vaccines versus correctly stored vaccines were also measured using the *Resolve* instrument. The data did not reveal any statistically significant features attributable to appearance or disappearance of chemical (vibrational) species in vaccine formulations (see Fig. S2). This suggest that SORS was not sensitive enough to capture subtle molecular changes in vaccine components following thermal exposure of vaccine vials.

Figure S2: (a) The results of the PCA analysis (PCA score plots) on SORS data acquired from thermally treated and correctly stored vaccines (red data points) using the *Resolve* instrument. (b) The relevant eigenvectors are also shown.

**S3: Comparison with Conventional Raman Spectroscopy**

Comparative measurements were also carried out through a, SII vaccine vial using a conventional handheld Raman instrument *TruScan* (Thermo Fisher Scientific USA). The results are summarised in Fig. S3. The tests show that conventional Raman spectroscopy is also capable of yielding well defined, low photon shot noise vaccine Raman signatures noninvasively. As such it is also a potential viable tool for differentiating falsified vaccines from genuine products in the field.

Although it is noted that the conventional Raman spectrum retains a significant interfering fluorescence background originating from vaccine glass vial [22] in the higher wavenumber region with *TruScan* spectra (~1300-1800 cm^-1^) (Fig. S3). Caution should be exercised when interpreting spectra in this spectral region as the fluorescence background, assumed to be due to glass impurities [22], presents a significant interfering feature as the relative intensity and position of these bands have been observed to vary from one glass type to another and can potentially also vary from one batch of glass to another preventing an effective subtraction of a single glass spectral profile obtained from a standard glass vial to be performed. As the underlying multiple fluorescence bands have bandwidth comparable to those of Raman features (e.g. water at ~1640 cm^-1^) their removal by fitting may also lead to the distortion of Raman spectra in this region (for example reducing unduly the intensity of the Raman band of water at ~1640 cm^-1^) a degree of which could vary with vial glass type and hampering potentially the ability of determining the concentration of or performing effective differentiation based around vaccine excipient concentrations.


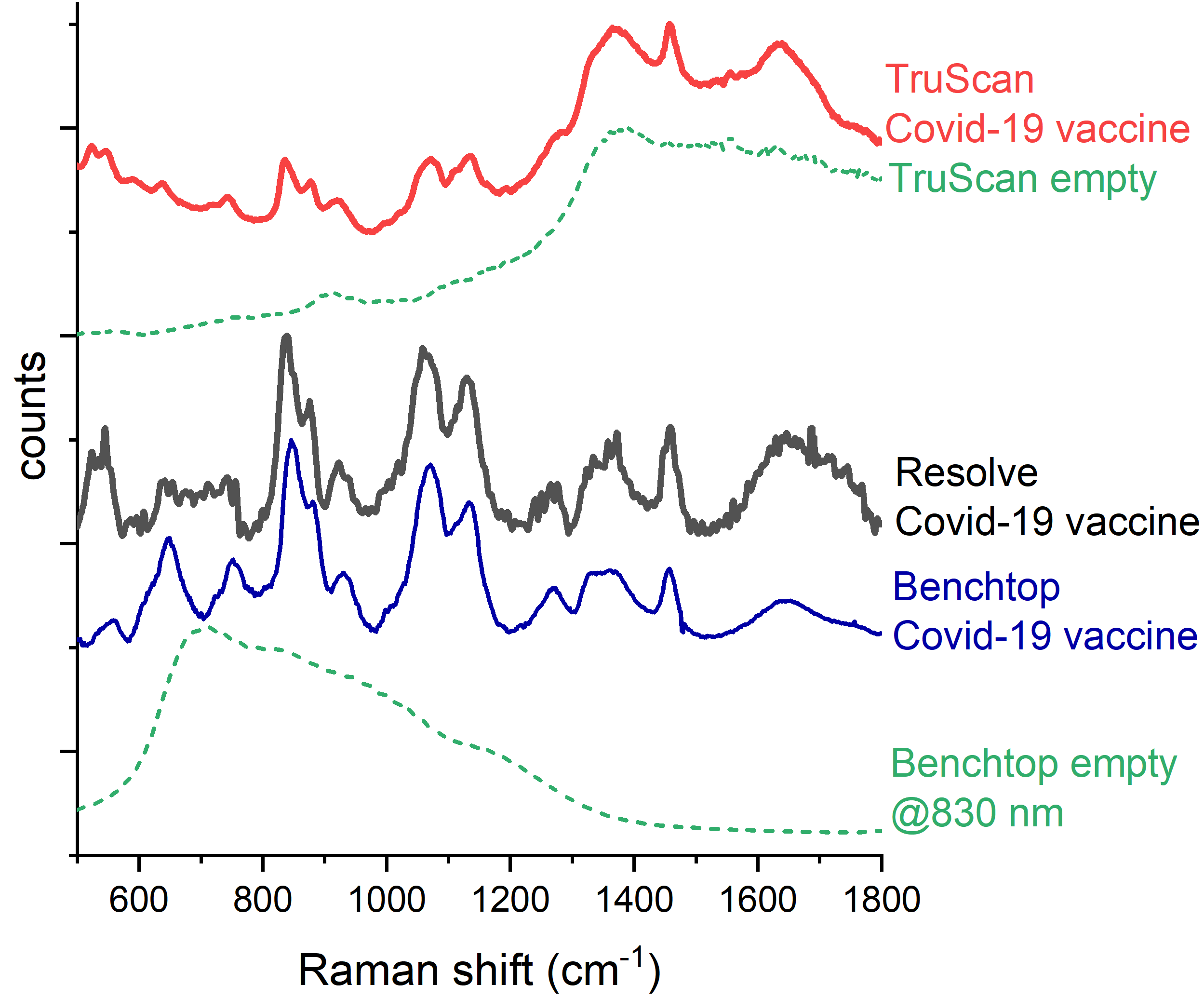


Figure S3: The comparison of the Raman spectra of SII vaccines measured through vaccine vials acquired using conventional Raman device *TruScan* (Raman excitation wavelength: 785 nm) and a SORS handheld device Resolve (Raman excitation wavelength: 830 nm) along with a spectrum obtained on a high-performance SORS benchtop instrument (Raman excitation wavelength: 830 nm) (blue). The green dashed lines are spectral profiles obtained from empty vials by conventional Raman instrument *TruScan* (top) and SORS benchtop instrument (bottom) set to conventional readout (zero spatial offset) illustrating the profile of interfering fluorescence signals originating from vial wall at each excitation wavelength. The fluorescence spectral feature is at a fixed absolute wavelength and as such it appears down shifted in Raman wavenumbers for the 830 nm Raman excitation spectra compared with that at 785 nm Raman excitation wavelength. The spectra are not corrected for intensity variation in the detection systems with wavelength.

The experimental conditions were as follows:

*Resolve*

The handheld SORS measurements were carried in the configuration described in the main body of in this paper. The Raman excitation wavelength was 830 nm and the laser power 475 mW. The acquisition time for the zero spatial offset was 5 s (1 s x 5 a) and 20 s (2 s x10 a) for the non-zero spatial offset.

*SORS* *Benchtop device*

The comparative benchtop measurements were carried out using a home-built, high performance SORS instrument described in detail elsewhere [^[[2]](#endnote-1)^]. The Raman excitation wavelength was 830 nm and the laser power 250 mW. The acquisition time was 100 s (2 s x 50 a) for the zero spatial offset and 100 s (2 s x 50 a) for the non-zero spatial offset (2 mm).

*TruScan*

The conventional Raman measurements were carried out using *TruScan RM* (Thermo Scientific). The Raman excitation wavelength was 785 nm and a laser power of 250 mW was used. The acquisition time was 20 s (1s x 20 a). A nose cone adaptor provided as an accessory was used as prescribed in operating manual.

The vials were measured in horizontal position through their bottom wall as previously for all the three devices.

1. †Present address: Hybrid Materials for Opto-Electronics Group, Department of Molecules and Materials, MESA+ Institute for Nanotechnology, Molecules Center and Center for Brain-Inspired Nano Systems, Faculty of Science and Technology, University of Twente, 7500AE Enschede, the Netherlands. [↑](#footnote-ref-1)
2. [] S. Mosca, P. Dey, T.A. Tabish, F. Palombo, N. Stone, P. Matousek, Anal. Chem. 91 (2019) 8994−9000. [↑](#endnote-ref-1)
